# A role for PDGF-C/PDGFRα signaling in the formation of the meningeal basement membranes surrounding the cerebral cortex

**DOI:** 10.1242/bio.017368

**Published:** 2016-03-17

**Authors:** Johanna Andrae, Leonor Gouveia, Radiosa Gallini, Liqun He, Linda Fredriksson, Ingrid Nilsson, Bengt R. Johansson, Ulf Eriksson, Christer Betsholtz

**Affiliations:** 1 Department of Immunology, Genetics and Pathology, Rudbeck Laboratory, Uppsala University, Uppsala 751 85, Sweden; 2 Department of Medical Biochemistry and Biophysics, Karolinska Institute, Stockholm 171 77, Sweden; 3 The Electron Microscopy Unit, Institute of Biomedicine, Sahlgrenska Academy at University of Gothenburg, Gothenburg 405 30, Sweden

**Keywords:** PDGF-C, Meninges, Basement membrane, Cerebrum, PDGFRα

## Abstract

Platelet-derived growth factor-C (PDGF-C) is one of three known ligands for the tyrosine kinase receptor PDGFRα. Analysis of *Pdgfc* null mice has demonstrated roles for PDGF-C in palate closure and the formation of cerebral ventricles, but redundancy with other PDGFRα ligands might obscure additional functions. In search of further developmental roles for PDGF-C, we generated mice that were double mutants for *Pdgfc^−/−^* and *Pdgfra^GFP/+^*. These mice display a range of severe phenotypes including spina bifida, lung emphysema, abnormal meninges and neuronal over-migration in the cerebral cortex. We focused our analysis on the central nervous system (CNS), where PDGF-C was identified as a critical factor for the formation of meninges and assembly of the glia limitans basement membrane. We also present expression data on *Pdgfa*, *Pdgfc* and *Pdgfra* in the cerebral cortex and microarray data on cerebral meninges.

## Abstract (precis)

**Summary:** Developmental expression of *Pdgfc* and *Pdgfra* is necessary for the development of meninges and the glia limitans basement membrane. Failure of these processes leads to neuronal over-migration.

## INTRODUCTION

Platelet-derived growth factors (PDGFs) and their receptors (PDGFRs) play pivotal roles in vertebrate development. Analyses of genetically modified mice have shown that three of the four mammalian PDGFs (PDGF-A, -B and -C), and both PDGFRs (PDGFRα and PDGFRβ) are important for a wide range of developmental processes, spanning from gastrulation to epithelial organogenesis, angiogenesis, hematopoiesis and other processes (reviewed by Andrae et al., 2008). For PDGF-D there is currently no published information available about its physiological role. PDGFs are dimeric polypeptides (PDGF-AA, -AB, -BB, -CC and -DD have been demonstrated to date) that act by binding to and inducing dimerization of PDGFRs in the plasma membrane, which in turn triggers receptor signaling. The PDGFRs are receptor tyrosine kinases (RTK) that signal via classical RTK pathways, including Ras-MAPK, PI3K and PLCγ (Heldin et al., 1998).

PDGFs differentially activate the PDGFRs. PDGF-C and PDGF-A are the principal ligands for PDGFRα *in vivo* as shown by gene knockout experiments: *Pdgfa^−/−^; Pdgfc^−/−^* double knockout mice phenocopy *Pdgfra^−/−^* mice (Ding et al., 2004; Soriano, 1997), whereas single *Pdgfa^−/−^* or *Pdgfc^−/−^* knockouts both display substantially milder phenotypes. This suggests that PDGF-C and PDGF-A exert partially overlapping and redundant functions via PDGFRα. *Pdgfb^−/−^*mice phenocopy *Pdgfrb^−/−^* mice (Hellström et al., 1999; Levéen et al., 1994; Soriano, 1994), demonstrating that PDGF-B is the principal physiological ligand for PDGFRβ. Despite the fact that PDGF-B can activate also PDGFRα *in vitro*, no redundancy between PDGF-A/C and PDGF-B has been implicated through comparative analysis of knockout mutants so far. However, PDGF-B may have physiological functions via PDGFRα that have gone unnoticed. Likewise, although PDGF-D can activate PDGFRβ *in vitro*, the physiological role of this ligand remains to be elucidated. Thus, even though much is known about the developmental roles of PDGFs through the analysis and comparison of individual knockout mice, the early embryonic lethality of some of the PDGF/PDGFR mutants and the likely redundancy between some of the PDGF ligands (in particular PDGF-C and PDGF-A) suggests that developmental functions exist that were not revealed through previous analyses.

The relative importance of different PDGFs for developmental processes may also vary depending on the genetic background, as illustrated by the perinatal lethality of *Pdgfc^−/−^* mice on a 129S1 background due to a complete cleft palate (Ding et al., 2004), whereas the same mutants survive into adulthood when bred on C57BL/6J background (Fredriksson et al., 2012). Thus, both redundancy with other PDGFs and genetic background may variably compensate for the loss of PDGF-C in *Pdgfc^−/−^* mice. The study of single or multiple *Pdgf* knockouts in different genetic backgrounds may therefore add further information about the developmental roles for PDGFs. Moreover, a reduction in PDGF receptor expression levels or signaling may sensitize mice to the loss of single PDGF ligand isoforms. Thus, a way to expose hidden roles of PDGF-C signaling via PDGFRα could be to reduce the level of PDGFRα expression in *Pdgfc^−/−^* mice.

Here, we adopted this strategy by generating *Pdgfc^−/−^; Pdgfra^GFP/+^* double mutants. Besides being a *Pdgfra* null allele, *Pdgfra^GFP^* offers the additional benefit of reporting cells that express *Pdgfra* through the expression of nuclear GFP. These studies demonstrate that complete loss of PDGF-C together with loss of a single functional copy of *Pdgfra* unmask previously unrecognized roles of PDGF-C in the brain and its surrounding meninges, the lungs and the vertebral column. These observations demonstrate that PDGF-C plays a role in lung and vertebral development, similar to what has previously been shown for PDGF-A. However, our analysis also reveals phenotypes not previously observed in PDGF/PDGFR mutants, demonstrating that PDGFRα signaling is required for the establishment of certain CNS compartments. Specifically, we noticed neuronal overmigration in the cerebral cortex. We observed that loss of PDGF-C signaling compromises the structure of cerebral meninges, which we hypothesize is the primary cause of the observed brain defects, since intact meninges are considered to be critical for normal CNS development (reviewed by Decimo et al., 2012; Siegenthaler and Pleasure, 2011).

## RESULTS

### *Pdgfc*^−/−^; *Pdgfra^GFP/+^* mice die perinatally

**Fig. 1.**
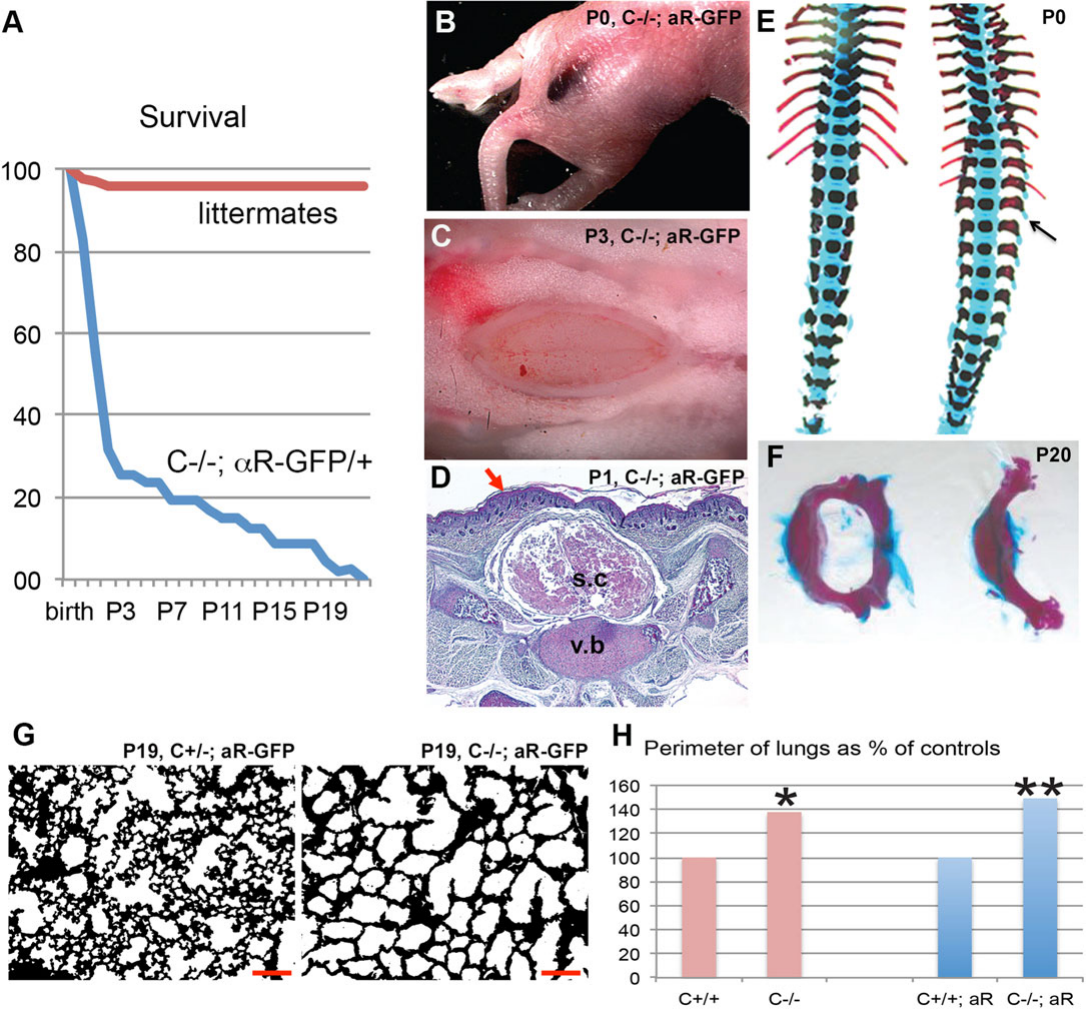
**Survival curve, spina bifida and lung phenotype of *Pdgfc^−/−^; Pdgfra^GFP/+^* mice.** (A) Kaplan–Meier curve showing survival of *Pdgfc^−/−^; Pdgfra^GFP/+^* pups and their littermates. Based on 47 *Pdgfc^−/−^; Pdgfra^GFP/+^* pups. (B) Newborn *Pdgfc^−/−^; Pdgfra^GFP/+^* mouse with haemorrhage over the dorsal back of the spine indicating a severe spina bifida. (C) Spina bifida after removal of the skin at P3. (D) Transverse section through the spina bifida area at P1, showing the presence of skin covering the affected area (arrow). s.c., spinal cord; v.b., vertebral body. (E) Alcian Blue/Alizarin Red staining of spinal column at P0, wild type (wt) (left) and *Pdgfc^−/−^; Pdgfra^GFP/+^* (right). Arrow marks an unfused vertebral arch. (F) Freely dissected lumbar vertebrae at P20, stained with Alcian Blue/Alizarin Red, wt (left) and *Pdgfc^−/−^; Pdgfra^GFP/+^* (right). (G) Paraffin section of P19 lung from *Pdgfc^+/−^; Pdgfra^GFP/+^* (left) and *Pdgfc^−/−^; Pdgfra^GFP/+^* (right). Note the absence of secondary alveolar septa in the mutant lung. Scale bar: 100 µm. (H) Quantification and comparison of perimeter of open areas in paraffin sectioned lungs. Open areas in *Pdgfc^−/−^* mice were significantly larger than *Pdgfc^+/+^* control mice, both in absence and presence of *Pdgfra^GFP/+^*. *P<0.05, **P<0.01.

*Pdgfc^−/−^* mice on a 129S1 background die at birth as a consequence of cleft palate (Ding et al., 2004), whereas they are viable and fertile on C57BL/6J background (Fredriksson et al., 2012). We generated *Pdgfc^−/−^; Pdgfra^GFP/+^* mice on C57BL/6J background, which were born from crosses of *Pdgfc*^+/−^; *Pdgfra^GFP/+^* and *Pdgfc*^+/−^ mice with a mendelian distribution [12% expected, 11.5% (19/165) were obtained]. In total we generated 74 *Pdgfc*^−/−^; *Pdgfra^GFP/+^* pups, out of which 47 were observed frequently and regularly until they suddenly died or were deemed necessary to euthanize for ethical reasons. The phenotype severity was variable and disease progression rapid, and although the mice were monitored daily, *Pdgfc^−/−^; Pdgfra^GFP/+^* mice that appeared vital in the evening were often found dead the following morning. During the first two days 72% of the *Pdgfc^−/−^; Pdgfra^GFP/+^* mice died or were euthanized, and only 14% were still alive at postnatal day (P)15. Two mice were followed until P21-22 (Fig. 1A).

At birth, *Pdgfc^−/−^; Pdgfra^GFP/+^* mice were easily distinguishable by the presence of a hemorrhagic stripe along the lower spine (Fig. 1B,C). These lesions were covered by an intact skin and, therefore, classified as spina bifida occulta (reviewed by Greene and Copp, 2009) (Fig. 1D). Indeed, we confirmed the presence of dorsally open vertebral arches in the lumbar region of *Pdgfc^−/−^; Pdgfra^GFP/+^* mice (Fig. 1E,F). This pronounced spina bifida occulta with associated local hemorrhage was exclusively observed in *Pdgfc^−/−^; Pdgfra^GFP/+^* mice and absent in all other littermates irrespective of their genotypes.

Similar to *Pdgfa* knockout mice (Boström et al., 1996), *Pdgfc^−/−^; Pdgfra^GFP/+^* mice displayed an emphysema-like phenotype in the lung (Fig. 1G). The average perimeter of the open airways was increased by 50% (*P*=0.0014) in *Pdgfc^−/−^; Pdgfra^GFP/+^* lungs, compared to *Pdgfc^+/+^; Pdgfra^GFP/+^* littermate controls. A similar but less severe phenotype was observed in *Pdgfc^−/−^* mice in comparison with *Pdgfc^+/+^*. We did not perform a side-by-side comparison to *Pdgfa^−/−^* mice, but on comparison with data reported earlier (Boström et al., 1996), the alveolar defects in *Pdgfc^−/−^* or *Pdgfc^−/−^; Pdgfra^GFP/+^* lungs appeared substantially milder than those in *Pdgfa^−/−^* mice.

### Cerebral abnormalities

**Fig. 2.**
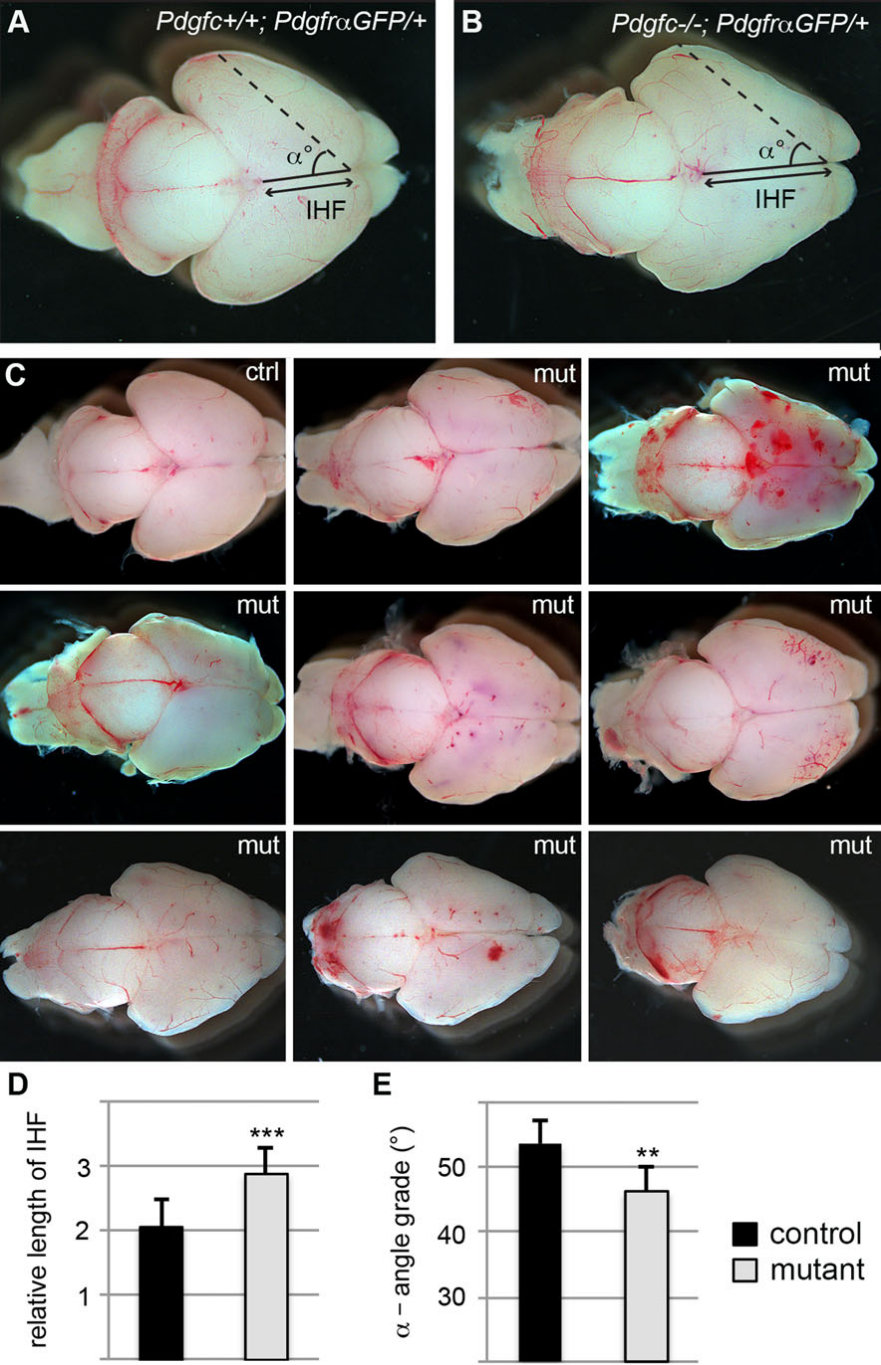
**Phenotypic and anatomic variations in brains of newborn mice.** Dorsal view of P0 brains from (A) *Pdgfc^+/+^; Pdgfra^GFP/+^* and (B) *Pdgfc^−/−^; Pdgfra^GFP/+^* mice. Filled line marks the interhemispheric fissure (IHF), the dotted line goes from the frontal end of the IHF to the most lateral point of the cerebrum. α is the angle between the two lines. (C) Examples of brains from newborn mice; one control (ctrl) and eight *Pdgfc^−/−^; Pdgfra^GFP/+^* (mut). Severity of both morphology and bleedings vary between the mutant mice. (D) Comparison of the length of the interhemispheric fissure in *Pdgfc^−/−^; Pdgfra^GFP/+^* (mut) and control mice. (E) Comparison of the angle α in *Pdgfc^−/−^; Pdgfra^GFP/+^* (mut) and control mice. Error bars show s.e.m.; **P<0.01, ***P<0.001.

Dissected brains from newborn *Pdgfc^−/−^; Pdgfra^GFP/+^* mice were clearly distinguishable from littermate control brains (Fig. 2). The cerebral hemispheres displayed an irregular shape and the cerebellum was reduced in size. To provide at least one quantitative measure of the cerebral abnormality, we assessed the interhemispheric fissure (IHF) and the angle (α) between then IHF and a line drawn from the frontal end of the IHF and the most lateral point of the cerebral hemisphere in newborn mice (Fig. 2A,B). Both were significantly different in *Pdgfc^−/−^; Pdgfra^GFP/+^* compared to littermate controls; IHF was 35% increased (*P*<0.0001; Fig. 2D) and α was 8% smaller (*P*<0.01; Fig. 2E).

**Fig. 3.**
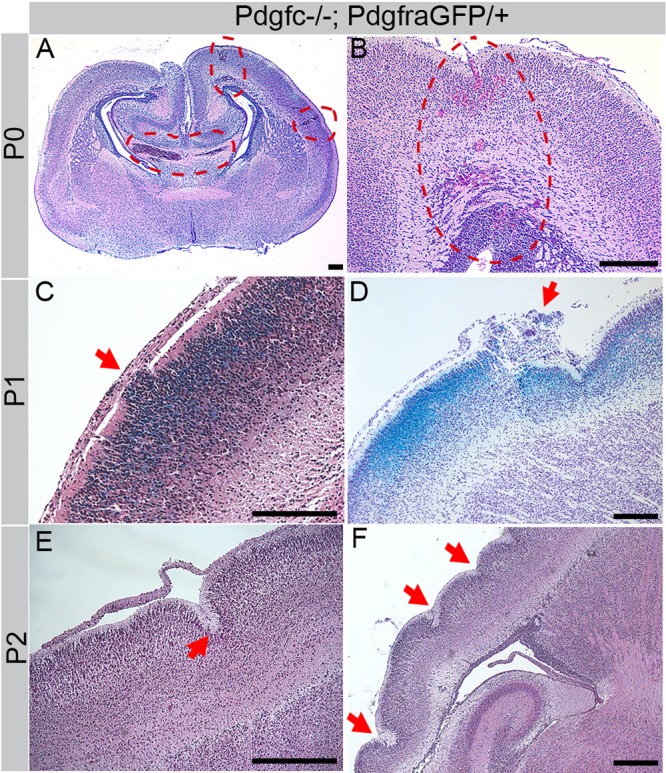
**Cerebral abnormalities in cortex of *Pdgfc^−/−^; Pdgfra^GFP/+^* mice.** Hematoxylin/eosin stained paraffin sections of mutant brains. (A) Coronal section at P0. Red dotted circles indicates areas of bleedings. (B) Coronal section, consecutive to previous image. Red dotted circle indicate bleedings all the way from the pial surface to the lateral ventricle. (C) Displaced cells (arrow) invade the marginal zone. Sagittal section of X-gal stained (blue) P1 brain. (D) Cresyl Violet stain at P1, sagittal section. Blue X-gal staining in cortical layer mark cells that normally express *Pdgfc*. Arrow indicates severely affected area. (E) Sagittal section at P2, arrow points at an invagination in the cortex. (F) Sagittal view of a wavy cortex at P2, arrows mark invaginations. Scale bars: 200 µm.

**Fig. 4.**
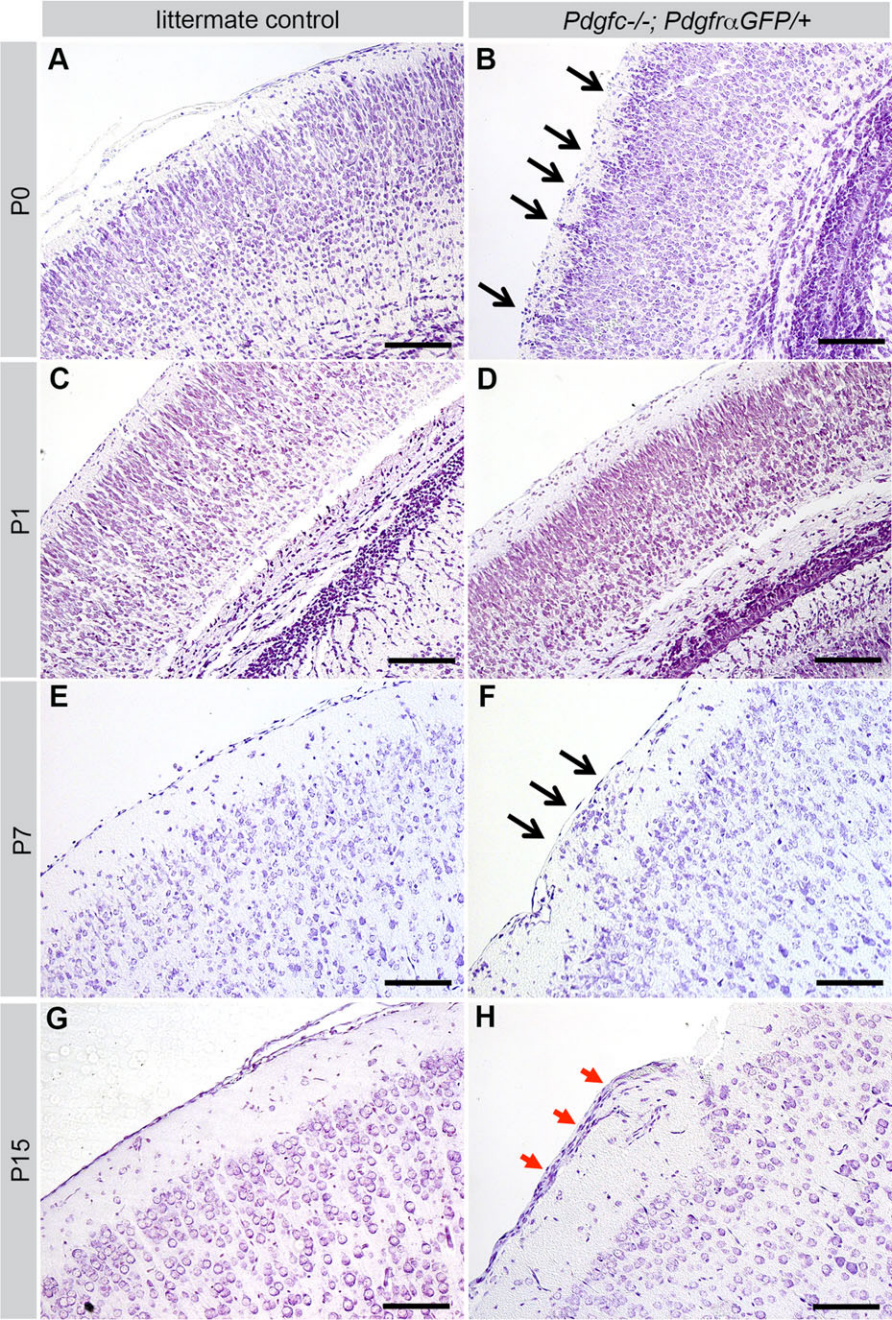
**Cerebral cortex at different postnatal ages.** Cresyl Violet staining of cerebral cortex of *Pdgfc^−/−^; Pdgfra^GFP/+^* (right) and littermate controls (left). A-F are coronal sections, G-H are sagittal. (A) *Pdgfc^+/−^; Pdgfra^GFP/+^* at P0. (B) Cortical neurons extend into the marginal zone (black arrows) of *Pdgfc^−/−^; Pdgfra^GFP/+^* at P0. (C) *Pdgfra^GFP/+^* at P1. (D) Thin cortex in *Pdgfc^−/−^; Pdgfra^GFP/+^* at P1. (E) *Pdgfc^+/−^; Pdgfra^GFP/+^* at P7. (F) Displacement of cells (black arrows) close to the meningeal border in *Pdgfc^+/−^; Pdgfra^GFP/+^* at P7. (G) *Pdgfra^GFP/+^* at P15. (H) Thick layer of cells outside of the marginal zone (red arrows) in *Pdgfc^−/−^; Pdgfra^GFP/+^* at P15. Scale bars: 150 µm.

Bleedings were frequently observed in *Pdgfc^−/−^; Pdgfra^GFP/+^* brains, both superficially (Fig. 2C) and deep in the brain parenchyma (Fig. 3A,B). The extent of the hemorrhage and regions involved varied between individuals. In tissue sections of newborn pups, extravasated erythrocytes were observed in the cerebral cortex, corpus callosum, midbrain, colliculus and cerebellum (Fig. 3A,B, circled areas). Other abnormal phenotypes observed included misplaced neurons (Fig. 3C,D) and folds in the cortical surface (Fig. 3E,F). These defects were focal and locations varied between individuals. However, the gross morphology of the cortical cell layers appeared normal for the most part (Fig. 4). Nissl staining of the cerebral cortex revealed that different cortical cell layers were present, correctly positioned in relation to each other, although in focal regions along the cortical surface cell nuclei were misplaced in the marginal zone below the meninges (black arrows in Fig. 4B,F). Occasionally, increased numbers of cells were identified along the meninges (red arrows in Fig. 4H). Due to the low postnatal survival and sudden death of mutant mice, we managed to retrieve only 11 postnatal brains older than P3 for histological analysis. Those were all at different ages (P4-P22), and no statistical analysis could be performed with this data.

### Expression of *Pdgfa* and *Pdgfc* in the developing cerebral cortex

**Fig. 5.**
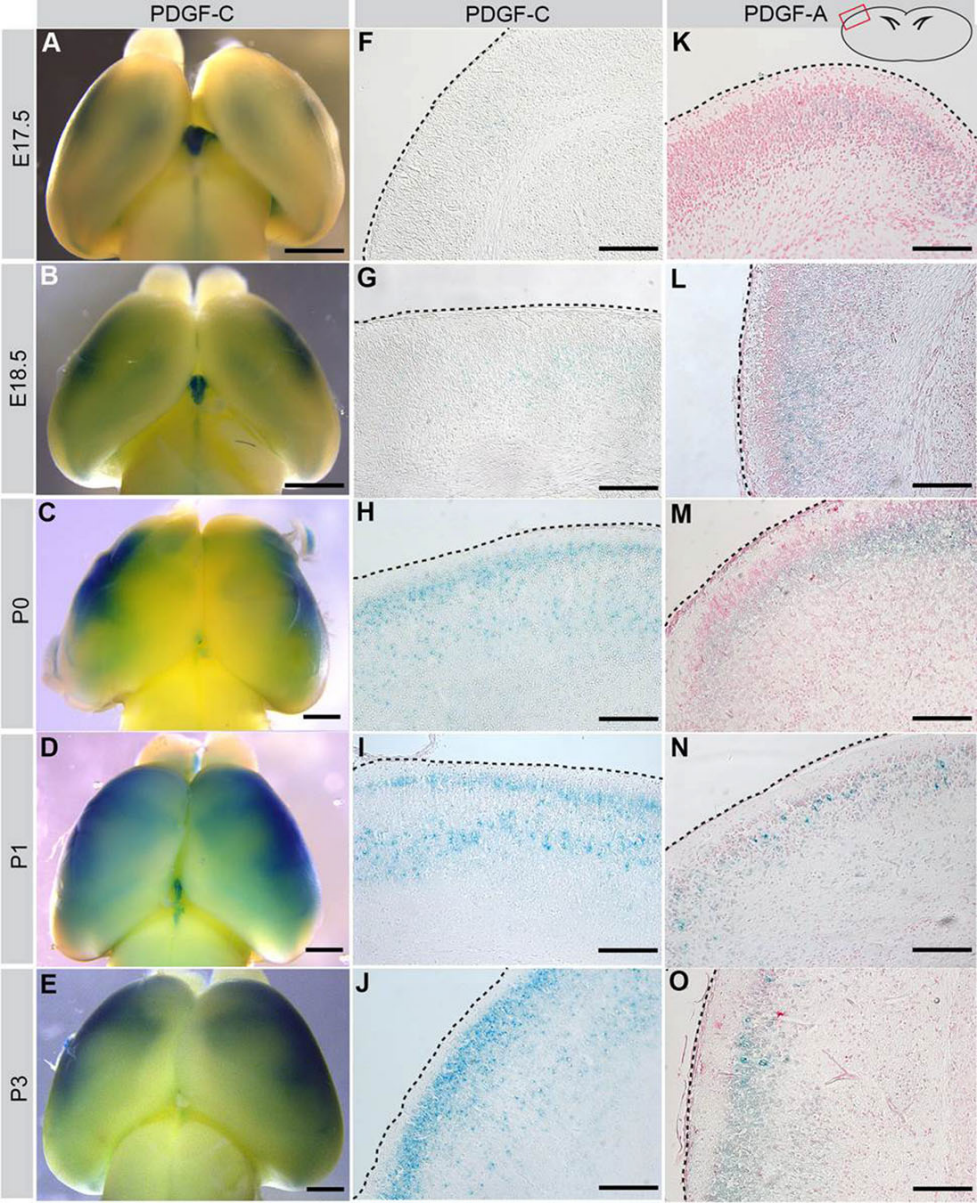
**Perinatal expression of PDGF-C and PDGF-A in cerebral cortex.** (A-E) Whole mount X-gal staining of *Pdgfc*^+/−^ brains, visualizing how PDGF-C expression increases with age. (F-O) Coronal sections of whole mount X-gal stained brains counterstained with Nuclear Fast Red. Illustration in upper right corner shows where the photos are taken, and black dotted lines mark the cortical surface. (F-J) Increasing expression of PDGF-C (blue) close to the cortical border. (K-O) PDGF-A expression (blue) largely overlaps with PDGF-C expression. Scale bar: 1 mm in A-E, 150 µm in F-O.

The cerebral defects in *Pdgfc^−/−^; Pdgfra^GFP/+^* mice imply an important role for *Pdgfra* signaling in the developing brain. We therefore analyzed the expression of PDGFRα and its main ligands PDGF-A, PDGF-C in the developing cerebral cortex at time points around birth [embryonic day (E)17.5, E18.5, P0, P1, P3]. As the *Pdgfa* and *Pdgfc* null alleles express *lacZ* from their respective promoters, we performed X-gal staining in heterozygous mice to localize sites of expression (Fig. 5).

During late embryonic development, PDGF-C was barely expressed in the forebrain, except in the choroid plexus in the lateral ventricles (Fig. 5A,B). PDGF-C expression first appeared in the dorsolateral cerebral cortex (Fig. 5B), and was intensified until P3 (Fig. 5A-E). Histological sections showed that expression was mostly confined to neurons in the cortical plate close to the glial border (Fig. 5F-J). The pattern of expression of PDGF-A was similar to that of PDGF-C (Fig. 5K-O). PDGF-C was not expressed in the skull covering the cerebral cortex at birth (data not shown).

Using *Pdgfra*^GFP/+^ mice, we mapped the expression pattern of PDGFRα-positive cells in the relevant areas of the cerebrum. The observed pattern is consistent with labeling of cerebral oligodendrocyte progenitors (OPCs) (Pringle et al., 1992). Before and early after birth, the numbers of PDGFRα-positive cells were low in the vicinity of the cortical surface, i.e. the regions where cellular abnormalities were observed, however, suggesting that the observed cerebral defects in *Pdgfc^−/−^; Pdgfra^GFP/+^* mice are not a result of defective signaling in OPCs.

### Abnormal cerebral meninges

**Fig. 6.**
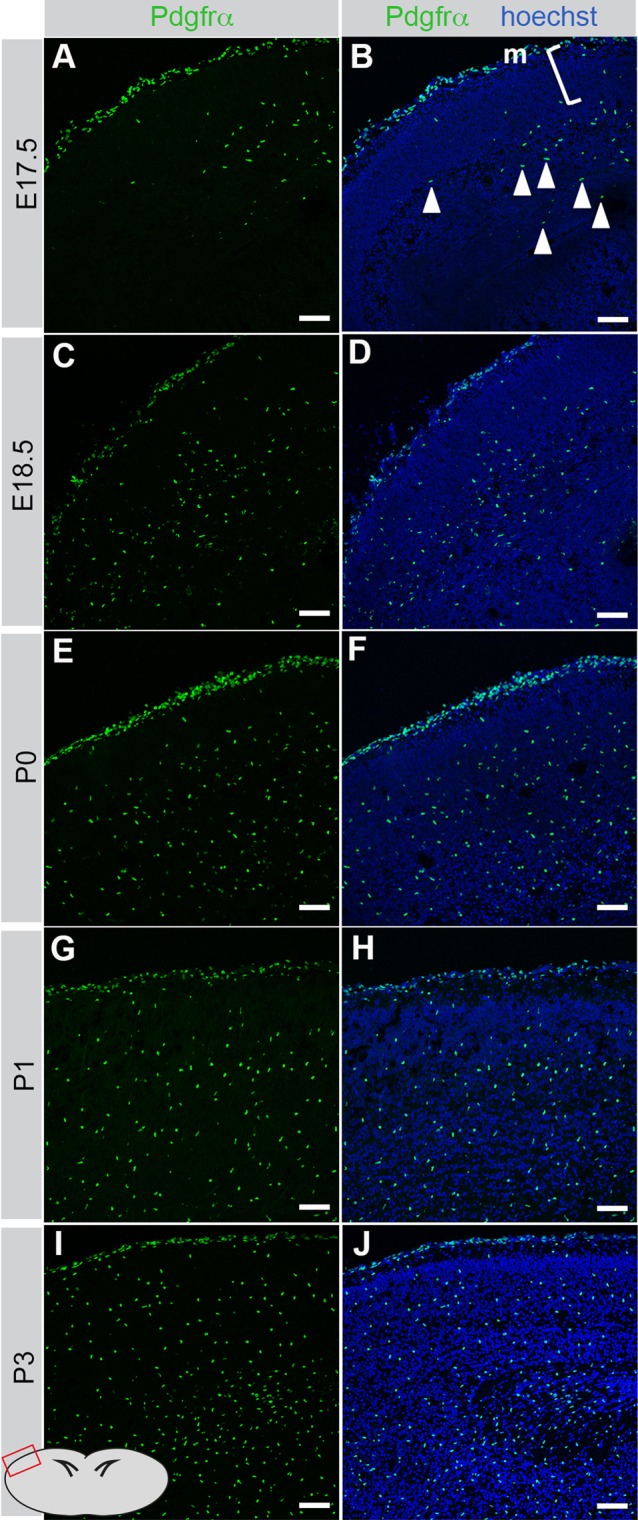
**Meningeal expression of PDGFRα in cerebral cortex.** Coronal sections from PDGFRα^GFP/+^ mice at E17.5 to P3. Illustration in lower left corner shows where the photos are taken. The majority of PDGFRα-GFP expressing cells were in the meninges (m) whereas very few cells were located in the underlying zone (marked [). Day by day, PDGFRα-positive oligodendrocyte precursors (arrowheads) populate the parenchyma. Scale bar: 60 µm.

**Fig. 7.**
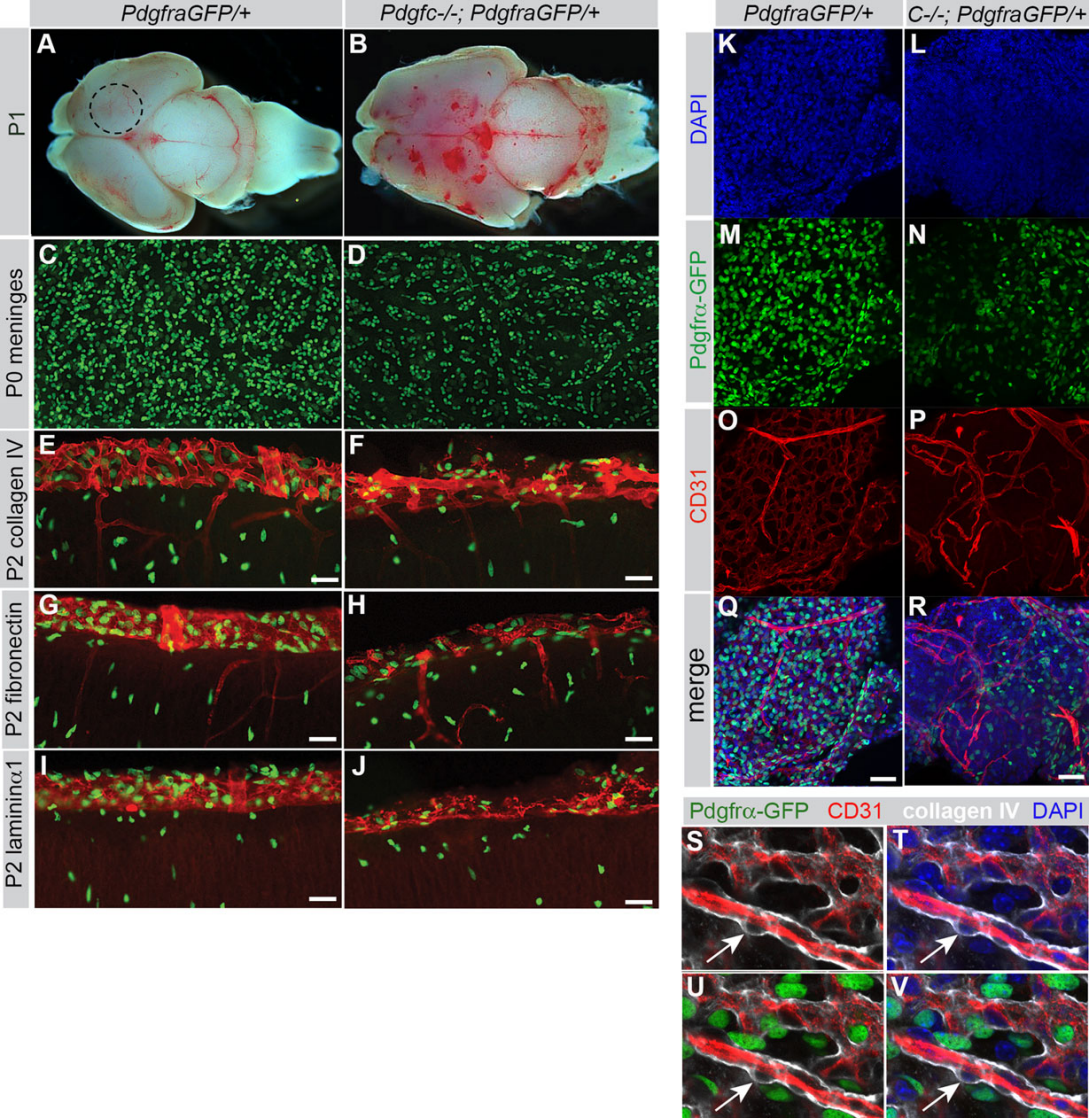
**Structural changes in cerebral meninges of *Pdgfc^−/−^; Pdgfra^GFP/+^* mice.** (A) Dorsal view of P1 brain from *Pdgfra^GFP/+^* mouse. Dotted circle indicates area for dissection of meninges. (B) Irregular shape and bleedings in brain from P1 *Pdgfc^−/−^; Pdgfra^GFP/+^* mouse. (C) Meninges peeled off from the dorsal, cerebral hemisphere of newborn *Pdgfra^GFP/+^* mice, dotted circle in A. (D) Reduced numbers of PDGFRα-positive cells in *Pdgfc^−/−^; Pdgfra^GFP/+^* meninges. (E-J) Immunofluorescent staining for extracellular matrix proteins Collagen IV (E,F), Fibronectin (G,H) and Laminin α1 (I,J) in coronal sections through the cerebral cortex in P2 pups show an irregular structure of the meninges in *Pdgfc^−/−^; Pdgfra^GFP/+^* mice. (K-R) Extended view of confocal *z*-stack of P4 meninges, *Pdgfra^GFP/+^* (left column) and *Pdgfc^−/−^; Pdgfra^GFP/+^* (right column). (K,L) DAPI. (M,N) Fewer PDGFR*α*-GFP-positive cells in mutants. (O,P) CD31 reveal variations in the vascular network. (Q,R) Merged view. (S-V) *Pdgfra* expression in non-vascular meningeal cells. Cerebral meninges from *Pdgfra^GFP/+^* brain immunostained for CD31 (red) and collagen IV (white). Arrow points at a PDGFRα negative vascular cell. Scale bars: 50 µm in E-J, 40 µm in K-R.

Since PDGFRα expression was high in the cerebral meninges (Fig. 6), we hypothesized that PDGFRα-positive meningeal cells could be target cells for PDGF-C expressed in the developing cerebral cortex. To analyze the meninges, we first peeled off meningeal sheets from the dorsal cerebrum (dotted circle in Fig. 7A), and mounted them on slides for *en face* morphological analyses. We found that *Pdgfc^−/−^; Pdgfra^GFP/+^* meninges were composed of fewer PDGFRα-GFP-positive cells than controls (Fig. 7C,D). Also, they were thin, fragile and more difficult to detach mechanically compared to meninges from control littermates. In coronal sections we verified that *Pdgfc^−/−^; Pdgfra^GFP/+^* cerebral meninges were indeed thinner and displayed an irregular pattern of extracellular matrix markers collagen IV, fibronectin and laminin α1 (Fig. 7E-J). These analyses revealed quantitative differences; all tested ECM molecules were present in mutant meninges, albeit in lower amounts.

As the superficial bleedings observed in *Pdgfc^−/−^; Pdgfra^GFP/+^* brains (Fig. 2C, Fig. 7B) were likely located within the meninges, we used the endothelial cell marker (CD31) to visualize vessels in meninges from the dorsal cerebrum. Blood vessels in *Pdgfc^−/−^; Pdgfra^GFP/+^* meninges were disorganized and significantly sparser compared to control mice (Fig. 7K-R). Meningeal cells are known to express PDGFRα (Pringle et al., 1992), but it is unclear which meningeal cell type(s) express it. Both CD31-positive endothelial cells and vascular mural cells, identified by their location between the collagen IV-positive vascular basement membrane and the CD31-positive endothelium were uniformly negative for PDGFRα. We conclude that in meningeal sheets from *Pdgfra^GFP/+^* mice, the nuclear GFP expression occurs in non-vascular mesenchymal cells (Fig. 7S-V).

### Neuronal gene expression in cerebral meninges

**Fig. 8.**
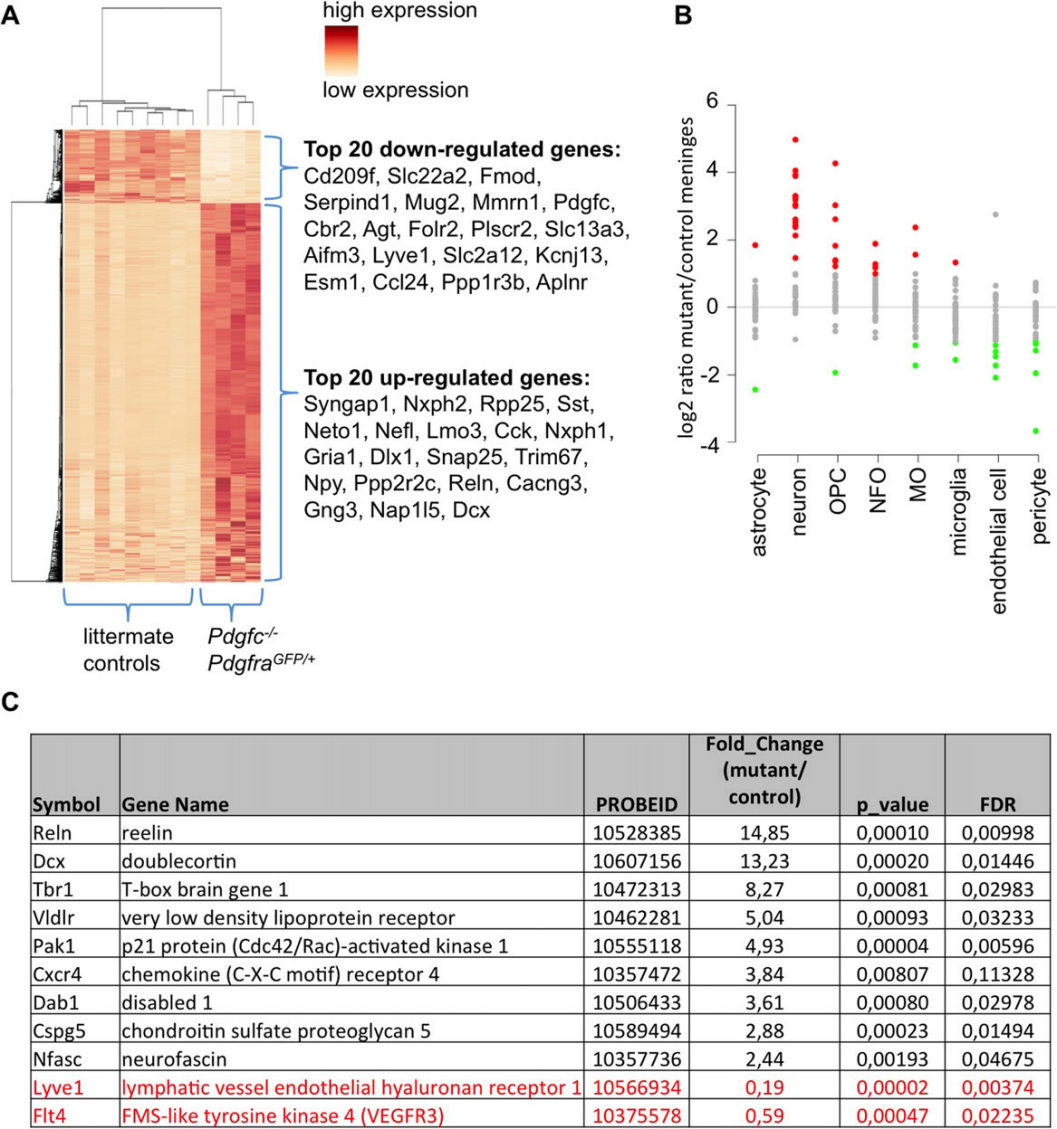
**Differentially expressed genes in cerebral meninges.** (A) Heat map showing the hierarchical clustering of differentially expressed genes in the meninges of *Pdgfc^−/−^; Pdgfra^GFP/+^* in comparison to littermate controls. Each row represents one gene and each column represents one animal. The expression levels are represented by color (dark color indicates high expression). The top 20 down- or upregulated genes are indicated to the right. (B) Gene expression changes in the top 40 cell-specific markers for eight different cell types, according to Zhang et al., (2014). Cell types are listed on the *x*-axis (OPC, oligodendrocyte precursor cell; NFO, newly formed oligodendrocyte; MO, myelinating oligodendrocyte). The *y*-axis represents the fold change (log_2_ scaled) between the mutants and the controls in meningeal samples. Each dot represents on gene (red, upregulated; green, downregulated; grey, no significant change). (C) Fold change, *P*-value and false discovery rate (FDR) of selected genes connected to neuron migration or lymphatic expression. Black genes are upregulated in mutant meninges; red genes are down regulated genes.

To gain additional insight into the basis for the morphological abnormalities in the meninges of the *Pdgfc^−/−^; Pdgfra^GFP/+^* mice, we isolated RNA from cerebral meninges of newborn mutants (*n*=4) and littermate controls (*n*=9) and analyzed the transcriptome using Affymetrix arrays. This analysis revealed extensive differences in gene expression between the two groups, with numerous genes being consistently up- or downregulated in the mutant meninges (Fig. 8A, www.ncbi.nlm.nih.gov/geo/, accession number GSE67644). The most unexpected finding was a highly significant upregulation of neuronal genes in cerebral meninges from *Pdgfc^−/−^; Pdgfra^GFP/+^* mice. Both KEGG and GO analysis confirmed high up-regulation of neuron-associated pathways (Table 1). The data was further compared to a transcriptome database for cell type-enriched genes in the central nervous system (Zhang et al., 2014) (Fig. 8B). We could then confirm that the genes upregulated in *Pdgfc^−/−^; Pdgfra^GFP/+^* meninges contained many representative markers for neurons and oligodendrocytes of different state of differentiation (Fig. 8B). In contrast, markers for vasculature, including endothelial and pericyte markers, were downregulated. The latter observation correlated with the decreased density of vasculature in mutant meninges (Fig. 7O,P). We also noticed upregulation of genes implicated in cortical neuronal migration, out of which some are listed in Fig. 8C. These are genes that, when mutated in mice, cause lissencephaly (Reln, Dcx, Vldlr, Dab1; reviewed by Olson and Walsh, 2002) or other abnormalities related to defective cortical neuronal migration during development; Cspg5 (Zhang et al., 2013), Cxcr4 (reviewed by Tiveron and Cremer, 2008); Pak1 (Pan et al., 2015) and Tbr1 (Hevner et al., 2001). Interestingly, we also noticed a strong decrease in expression of lymphatics markers in *Pdgfc^−/−^; Pdgfra^GFP/+^* meninges, which might implicate a role for PDGFRα in the development of meningeal lymphatics (Aspelund et al., 2015; Louveau et al., 2015).

**Table 1.**
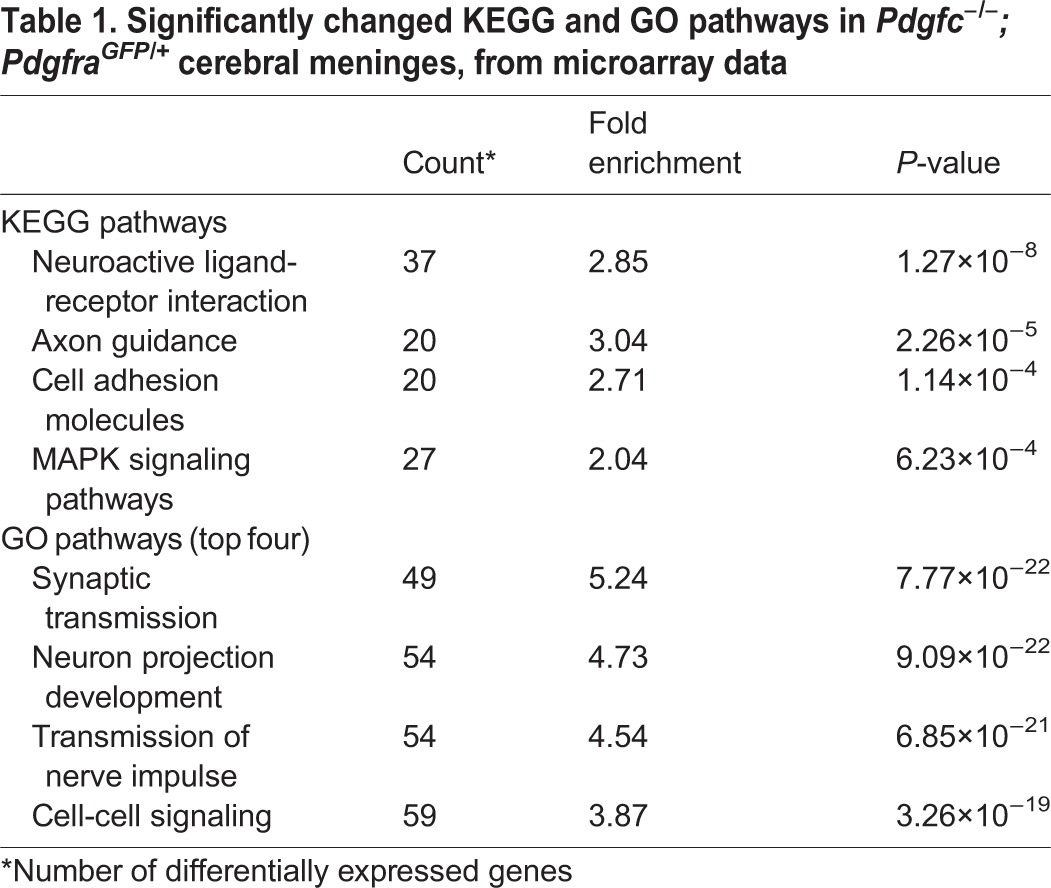
**Significantly changed KEGG and GO pathways in *Pdgfc^−/−^; Pdgfra^GFP/+^* cerebral meninges, from microarray data**

### Neuronal overmigration into compact meninges with discontinuous basement membrane coverage at the brain surface

**Fig. 9.**
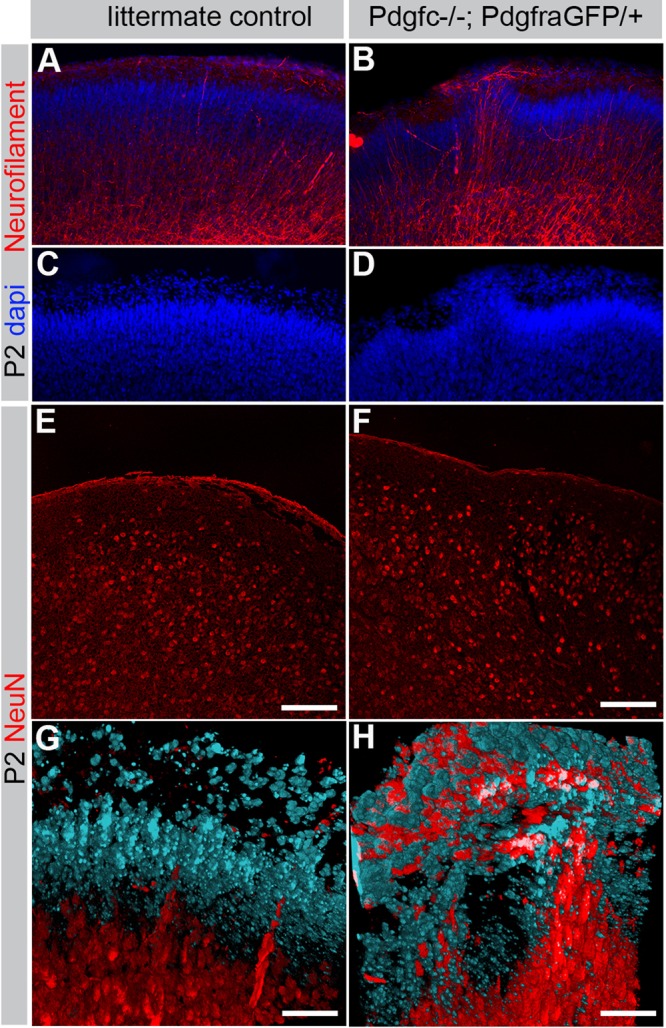
**Neuronal overmigration in P2 cerebral cortex.** (A,B) Immunofluorescent neurofilament staining (red) of coronal sections through the cerebral cortex visualizes neuronal overmigration in *Pdgfc^−/−^; Pdgfra^GFP/+^* mice. (C,D) Nuclear DAPI staining (blue) confirms displacement of the cortical cell layer in *Pdgfc^−/−^; Pdgfra^GFP/+^* mice. (E,F) Immunofluorescent staining of NeuN in coronal section without overmigration. (G,H) Maximum intensity projection of confocal *z*-stack visualizing cortical neurons with NeuN (red) and non-neuronal cells in the marginal zone with DAPI (blue). Scale bars: 100 µm in E,F; 50 µm in G,H.

Although recent data show that a small number of neural precursor cells reside within the meninges (Bifari et al., 2015, 2009), the meninges are normally devoid of mature nervous tissue. However, we hypothesized that the meninges from *Pdgfc^−/−^; Pdgfra^GFP/+^* mice could be contaminated with neurons as a result of neuronal overmigration from the brain parenchyma. Indeed, staining for neurofilaments provided overt signs of neuronal overmigration. Neurofilament-positive fibers stretched through the marginal zone into the meninges (Fig. 9A-D). A similar pattern was seen for NeuN-positive neuronal nuclei (Fig. 9G,H), indicating the translocation of entire neuronal cells into the meninges. Like the above-mentioned phenotypes, these signs of neuronal overmigration only appeared as focal lesions with variable locations; in most areas the NeuN-positive cell nuclei resided normally positioned within the cortical plate (Fig. 9E,F).

**Fig. 10.**
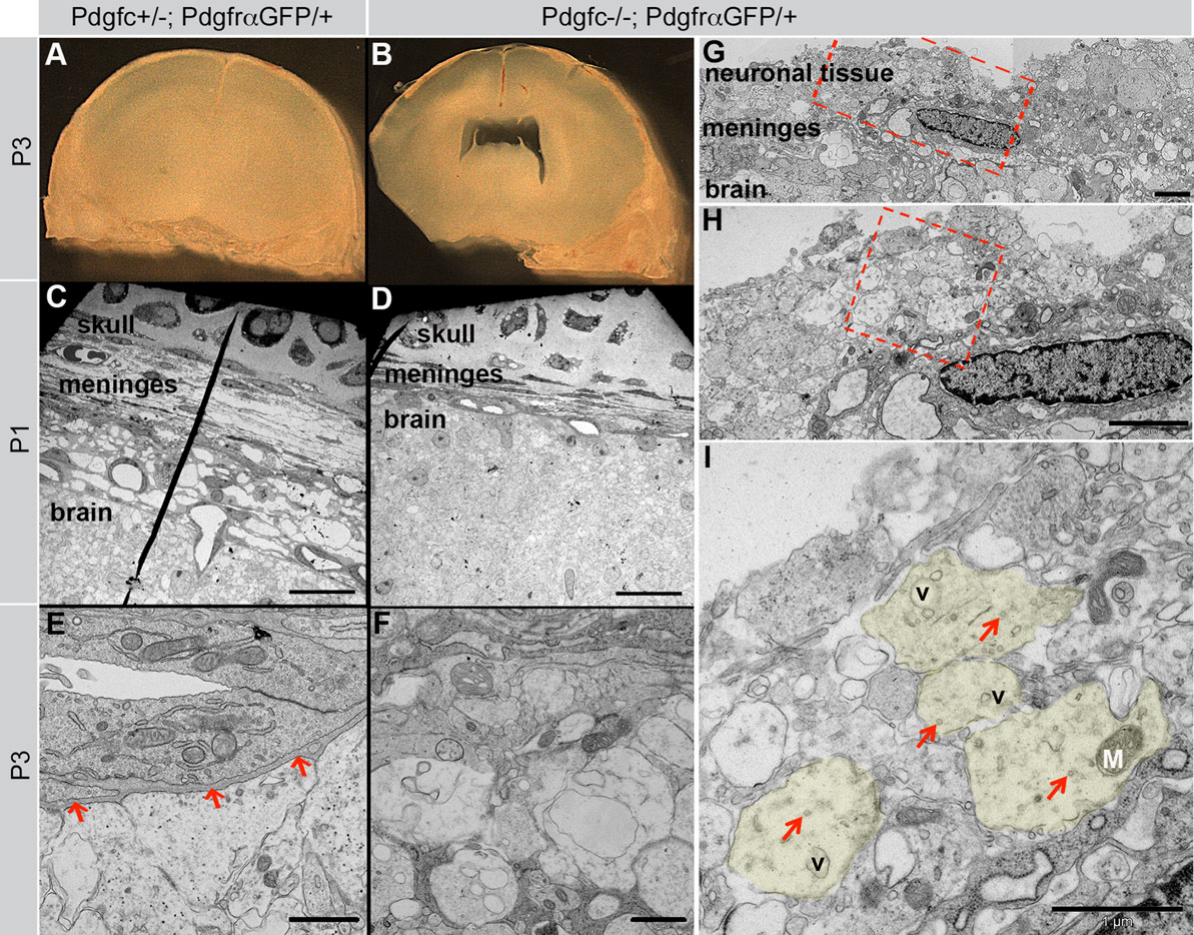
**Confirmation of neuronal overmigration in cerebral cortex.** (A,B) P3 vibratome sections prepared for TEM studies. The asymmetric lateral ventricles (seen in B) confirm data from Fredriksson et al. (2012). (C-I) Structural analysis with electron microscopy of cerebral meninges from *Pdgfc^−/−^; Pdgfra^GFP/+^*and *Pdgfc^+/−^; Pdgfra^GFP/+^* littermate control mice. (C) P1 cerebral meninges. (D) P1 mutant meninges are thin and condensed. (E) High magnification of the area of glia limitans and its basement membrane, in a P3 control mouse. Arrows indicate the basement membrane. (F) Area of glia limitans in a mutant P3 brain, no basement membrane is visible. (G) Neuronal tissue outside the meninges confirms neuronal overmigration in a mutant mouse. Red rectangle is magnified in H. (H) Neuropil outside the meninges. Red square is magnified in I. (I) High magnification of cross cut unmyelinated neurites (out of which some have been colored in light green). V marks vesicles, M marks a mitochondrion and arrows mark microtubule. Scale bars: 20 µm in C,D; 2 µm in E-H; 1 µm in I.

Neuronal overmigration could explain both why *Pdgfc^−/−^; Pdgfra^GFP/+^* meninges appeared more adhesive to the cerebrum and easily broke during dissection, as well as the observation of neuronal genes in the meninges. To further verify the neuronal overmigration, we performed transmission electron microscopy (TEM) analyses on meninges from *Pdgfc^−/−^; Pdgfra^GFP/+^* and *Pdgfc^+/−^; Pdgfra^GFP/+^* mice. We first made coronal vibratome sections from P1 and P3 brains with the skull still attached (Fig. 10A,B). In these preparations, we observed asymmetric enlargements of the lateral ventricles in *Pdgfc^−/−^; Pdgfra^GFP/+^* mice, in agreement with a previously reported defect in *Pdgfc^−/−^* mice (Fredriksson et al., 2012). The vibratome sections were trimmed in order to allow ultrathin cross sections of the cerebral meninges. TEM analysis showed that control meninges (*Pdgfc^+/−^; Pdgfra^GFP/+^*) were regularly formed of loosely packed tissue and voluminous extracellular matrix (Fig. 10C). Several cell layers were visible and a distinct basement membrane bordered the brain surface (arrows in Fig. 10E). In marked contrast, meninges in *Pdgfc^−/−^; Pdgfra^GFP/+^* mice were occasionally extremely thin and compact, with markedly reduced extracellular matrix volume (Fig. 10D). The most conspicuous specific abnormality of the *Pdgfc^−/−^; Pdgfra^GFP/+^* meninges was an indistinct, and in some areas totally missing, basement membrane (Fig. 10F). In the latter regions neuronal tissue was observed extending from within the neuropil into the meningeal arachnoid layer (Fig. 10G-I). These observations were consistent with neuronal overmigration.

### Involvement of other *Pdgf* genes

*Pdgfc^−/−^* mice develop a mild form of spina bifida (Ding et al., 2004), but homozygous *Pdgfc* knockout alone was not sufficient to induce the severe form of spina bifida occulta associated with hemorrhage that we observed in *Pdgfc^−/−^; Pdgfra^GFP/+^* mice (Fig. 1B). As an additional loss of one functional copy of the *Pdgfra* gene was also required to induce severe spina bifida occulta, we hypothesized that PDGF-C acts in concert with (an)other PDGF ligands in the formation of the vertebral arch. To identify this ligand, we generated *Pdgfc*^−/−^ mice that were also heterozygous for *Pdgfa, Pdgfb* or *Pdgfd*. The only genotype that resulted in a spina bifida occulta with hemorrhage was *Pdgfc^−/−^; Pdgfa^+/−^*, confirming recently published observations (Andrae et al., 2013). Neither *Pdgfc^−/−^; Pdgfb^+/−^* nor *Pdgfc^−/−^; Pdgfd^+/−^* showed any similar phenotype (Table 2). Tissue plasminogen activator (tPA) activates the latent PDGF-C protein *in vitro* (Fredriksson et al., 2004) and acts upstream of PDGF-C/PDGFRα in regulation of blood brain barrier integrity *in vivo* (Su et al., 2008). To investigate if loss of tPA would phenocopy loss of PDGF-C during vertebral formation, we generated *tPA*^−/−^; *Pdgfra^GFP/+^*. Also this combination failed to reproduce spina bifida, suggesting that other protease(s) than tPA can activate PDGF-C during mouse development.

**Table 2.**
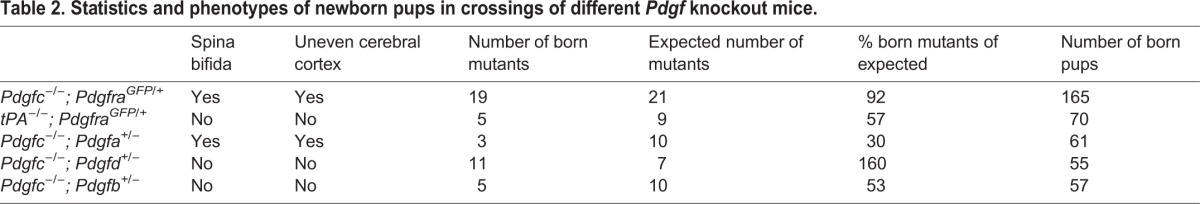
**Statistics and phenotypes of newborn pups in crossings of different *Pdgf* knockout mice.**

## DISCUSSION

### Study rationale

Analysis of *Pdgfc^−/−^* mice has already demonstrated that PDGF-C play roles in palate closure and the formation of CNS ventricles (Ding et al., 2004; Fredriksson et al., 2012). However, redundancy between PDGF-C and other PDGFs that signal via PDGFRα may hide other functions. We reasoned that the deletion of one copy of the *Pdgfra* gene, which alone has no known phenotypic consequences, could synergize with *Pdgfc* deficiency and unmask hitherto hidden functions. The *Pdgfra^GFP^* knock-in allele (Hamilton et al., 2003) is a null allele that expresses GFP from the endogenous *Pdgfra* promoter. Homozygous *Pdgfra^GFP/GFP^* mice are embryonically lethal like *Pdgfra^−/−^* mice (Soriano, 1997). We found that *Pdgfc^−/−^; Pdgfra^GFP/+^* mice develop multiple abnormalities, some of which confirmed previously reported phenotypes, whereas others were novel and surprising. Both PDGF-C and its receptor PDGFRα are broadly expressed in developing and adult mammals (Aase et al., 2002; Orr-Urtreger and Lonai, 1992). A complex and lethal phenotype was therefore expected, but the range of observed defects (e.g. in brain, spine, lung and vasculature) made it difficult to pinpoint a single cause of death. Phenotype severity and age of lethality was also variable despite a homogenous C57BL/6J background.

### Differential importance of PDGF-C and PDGF-A in vertebral, lung and oligodendrocyte development

Some of the phenotypes observed in *Pdgfc^−/−^; Pdgfra^GFP/+^* mice confirm previous observations. One of them, spina bifida occulta, implies that the vertebral arches are not properly closed. Spina bifida is a common developmental defect in humans with numerous underlying causes, as well as a diverse range of consequences, depending on the severity (reviewed by Greene and Copp, 2009). Vertebral arches are normally formed from the axial mesoderm that migrates dorsally to cover the closed neural tube. In open spina bifida there is also a problem with the closure of the neural tube itself; as a result, the axial mesoderm cannot cover the unclosed area leading to defective formation of the vertebral arches. In this case, the neural tube is either left openly exposed or covered by meninges. Spina bifida occulta (hidden spina bifida) is a milder variant where the problem resides in the axial mesoderm itself. The vertebral arches form abnormally also in this case, but the lesion is covered with skin and sometimes undetectable in mice without invasive examination (i.e. dissection). Severe spina bifida occulta has previously been observed in *Pdgfra* null and signaling-deficient mouse mutants (Hamilton et al., 2003; Klinghoffer et al., 2002; Soriano, 1997) and in *Pdgfa; Pdgfc* double knockout mutants (Ding et al., 2004). Spina bifida occulta with variable severity and penetrance has also been observed in *Pdgfc^−/−^*, *Pdgfc^−/−^;Pdgfa*^Δex6/Δex6^, and *Pdgfc^−/−^;Pdgfa*^+/−^ mice (Andrae et al., 2013; Ding et al., 2004), but not in single *Pdgfa* knockouts. These results suggest that PDGF-C plays a critical role in vertebral development that is overlapping and partially redundant with PDGF-A. The severe and fully penetrant spina bifida occulta observed here in *Pdgfc^−/−^; Pdgfra^GFP/+^* mice suggest that this overlapping function is mediated by PDGFRα.

Whereas PDGF-C seems to be the most important PDGFRα ligand in vertebral development, PDGF-A appears to be the most important PDGFRα ligand in lung development. A severe lung emphysema-like phenotype has been reported in *Pdgfa*^−/−^ mice (Boström et al., 1996; Lindahl et al., 1997) and a similar but milder lung phenotype was observed in *Pdgfa*^Δex6/−^*; Pdgfra^GFP/+^* mice (Andrae et al., 2013). The emphysema-like phenotype observed here in *Pdgfc^−/−^; Pdgfra^GFP/+^* and in *Pdgfc^−/−^* mice, shows that PDGF-C indeed plays a role in lung development, but PDGF-C is clearly less important in this organ compared to PDGF-A.

The numbers of PDGFRα-positive oligodendrocyte precursors in the spinal cord depend directly on the concentration of PDGF-A, and *Pdgfa*^−/−^ mice develop oligodendrocyte hypoplasia and hypomyelination (Andrae et al., 2013; Calver et al., 1998; Fruttiger et al., 1999). Loss of PDGFRα-positive oligodendrocyte progenitors has also been observed in *Pdgfra* null and signaling deficient mice (Calver et al., 1998; Fruttiger et al., 1999; Klinghoffer et al., 2002). PDGF-C is expressed in the developing CNS, and could therefore be partially redundant with PDGF-A in oligodendrocyte development. However, when we performed a quantitative analysis of *Pdgfra^GFP^* expressing cells in the E15.5 spinal cord of *Pdgfc^−/−^; Pdgfra^GFP/+^* and *Pdgfra^GFP/+^* embryos, there was no correlation between PDGF-C expression and the number of PDGFRα-positive cells (data not shown). These results suggest that PDGF-C plays no or only a minor role in oligodendrocyte development.

### Neuronal overmigration

*Pdgfc^−/−^; Pdgfra^GFP/+^* mice displayed neuronal overmigration, verified by immunofluorescent staining for neuronal markers, transmission electron microscopy and transcriptional profiling of meningeal tissues. The results from the microarray analyses were highly consistent, showing multiple neuron-associated genes being upregulated in *Pdgfc^−/−^; Pdgfra^GFP/+^* meninges. Recent publications have suggested that meninges harbor a set of neural precursor cells (Bifari et al., 2009, 2015), expressing, for example, doublecortin (*Dcx*). *Dcx* was upregulated also in *Pdgfc^−/−^; Pdgfra^GFP/+^* meninges, which may suggest an increase in neural precursor cells, but the upregulated genes in the *Pdgfc^−/−^; Pdgfra^GFP/+^* meninges included multiple genes normally expressed by mature neurons, such as Tubb3 (Tuj1), which is not expressed by meningeal neural precursor cells (Bifari et al., 2015).

**Fig. 11.**
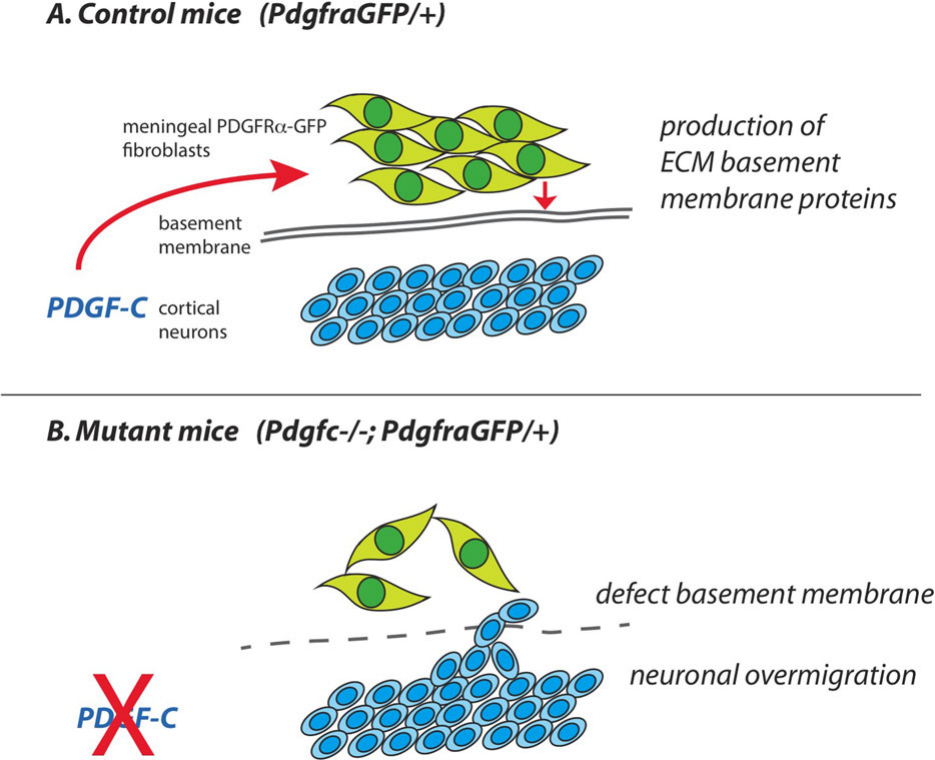
**Suggested model.** (A) Based on data in this paper we suggest that PDGF-C expressed by neurons in the cerebral cortex is needed for proper organization of PDGFRα-positive meningeal fibroblasts, production of extracellular matrix proteins (ECM) and formation of the glial basement membrane. (B) Without PDGF-C, the arrangement of meningeal cells and ECM is irregular, no proper basement membrane is formed and cortical neurons can migrated out of the brain.

Based on the pattern of expression of PDGF-C and PDGFRα in the developing brain and associated meninges, we suggest that PDGF-C, expressed by neurons in the neocortex, signal to PDGFRα-positive cells in the meninges (Fig. 11). The abnormalities and regional loss of basement membrane integrity at the brain surface offers a plausible explanation for the observed neuronal overmigration in *Pdgfc^−/−^; Pdgfra^GFP/+^* mice. Previous work demonstrates that changes in meningeal gene expression patterns may result in brain abnormalities. Neuronal migration and interaction with the meninges are important for correct cerebral cortex development, and different signaling pathways are involved. Defective CXCL12/CXCR4 signaling (Borrell and Marín, 2006) and hypomorphic *Foxc1* (Zarbalis et al., 2012) generate defective forebrain meningeal formation, which, in turn, impairs tangential migration of cortical interneurons and Cajal Retzius cells. The *Pdgfc^−/−^; Pdgfra^GFP/+^* mice and *Foxc1* hypomorphic mice (Zarbalis et al., 2007) share phenotypic abnormalities, including defective meninges and neuronal overmigration. The major cortical dysplasias in *Foxc1* mice develop the first week after birth, which could not be studied in *Pdgfc^−/−^; Pdgfra^GFP/+^* mice because the most severe cases died perinatally. Interestingly, *Pdgfra* signaling was recently placed downstream of *Foxc1* in zebrafish development (French et al., 2014).

### A primary defect in the meninges

During CNS development, PDGFRα expression is mainly restricted to glial cells, starting at E12.5 in oligodendrocyte precursors in the ventral spinal cord (Pringle and Richardson, 1993). There are also reports on PDGFRα expression in early neuroepithelial cells and postnatal cerebellar neurons (Andrae et al., 2001; Nait Oumesmar et al., 1997). In the meninges surrounding the brain, we found that PDGFRα expression was abundant at all analyzed stages. The exact identity of the PDGFRα-positive cells in meninges is not known. Generally, PDGFRα expression in different organs is localized to fibroblasts and other types of mesenchymal cells. Here, we confirm PDGFRα expression in meningeal cells that were not vascular-associated, but likely of mesenchymal origin. Yet, additional analyses are needed to assign the PDGFRα-positive cells to any (or several) specific cell type or category. The structural and cellular composition of the murine meninges is not well elucidated. Most histological analyses on meninges have been done on human material. Grossly, three different layers make up the meninges: (1) the innermost pia mater, a fibrous layer of flat cells separated from the brain by a basement membrane, (2) the arachnoid, a collagen-rich layer that partly forms a trabecular network containing blood vessels and (3) the outermost dura mater, a harder layer of thick connective tissue attached to the skull (Haines et al., 1993). The pia, arachnoid and the intervening sub-arachnoid space containing blood vessels are often referred to as the leptomeninges. Meninges form early in development, in mice already at E9-10 (reviewed by Siegenthaler and Pleasure, 2011).

It is becoming increasingly evident that the meninges and their basement membranes play a crucial role in the patterning of the CNS. Work by Sievers and colleagues suggest that an impaired meningeal layer affects cerebellar foliation (Sievers et al., 1981). Further work suggested that fibroblast-like meningeal cells deposit extracellular matrix proteins involved in the formation of the glia limitans (Sievers et al., 1994). Additional reports describe molecules and signaling pathways that influence formation of the meningeal basement membrane, the disturbance of which negatively influences both cerebellar and cerebral patterning. For example, mice lacking β1 integrin in neuronal- and glial precursor cells die prematurely with defective basement membrane and glial end-feet formation, irregular cerebral cortical lamination, neuronal overmigration, smaller cerebellar folia and distorted laminar organization (Graus-Porta et al., 2001). This study suggested that the neuron/glia interaction with the meningeal basement membrane is needed for maintenance/remodeling of ECM deposited by meningeal cells. Complete ablation of α6 integrin results in meningeal basement membrane gaps, leading to cortical lamination defects in the developing cerebral cortex (Georges-Labouesse et al., 1998). These data were not reproduced by tissue specific deletion of a6 integrin in neuronal and glial precursor cells, however, suggesting a primary defect in the meninges (Marchetti et al., 2013). A6β1 integrin is a receptor for the adhesion molecule laminin, which is also important for cerebellar development by affecting the meningeal basement membrane.

Intrinsic meningeal defects may also explain the vascular abnormalities observed in *Pdgfc^−/−^; Pdgfra^GFP/+^* mice, with superficial as well as intracranial bleedings. Although PDGFRα-positive meningeal cells were not identified as endothelial or mural cells, the meningeal vessels in mutants were uneven and irregular, and the expression of vascular-associated genes was reduced in *Pdgfc^−/−^; Pdgfra^GFP/+^* meninges. These results confirm and extend previously described effects of PDGF-C on vascular development and revascularization (Fredriksson et al., 2012; Moriya et al., 2014). Recently, lymphatics were identified in murine meninges (Aspelund et al., 2015; Louveau et al., 2015). We found that both Lyve1 and VEGFR3 were downregulated in *Pdgfc^−/−^; Pdgfra^GFP/+^* meninges possibly implicating that meningeal lymphatics may be disturbed in these mice. This issue will need further investigation.

The structure and formation of the murine meninges is a relatively unexplored area, and there are many interesting paths to elucidate further. Here, we show that signaling of PDGF-C is necessary to form an intact meningeal layer around the mouse cerebrum, and we propose that this leads to secondary developmental brain defects. What kind of cells that express PDGFRα, their cooperation with other cells and contribution to the extra cellular matrix to meningeal basement membrane assembly are questions that warrant further study.

## MATERIALS AND METHODS

### Mouse strains

All mice were bred according to Swedish animal welfare legislation. The National ethical committee approved the project. Mouse strains in use: *Pdgfa* knockout (Boström et al., 1996); *Pdgfa^ex4COIN−INV-lacZ^* (Andrae et al., 2014)*; Pdgfb* knockout (Lindahl et al., 1997); *Pdgfc* knockout (Ding et al., 2004); *Pdgfd* knockout (H. Gladh et al., Karolinska Institutet, Sweden, unpublished); *Pdgfra^GFP^* (Hamilton et al., 2003); *tPA* knockout (Carmeliet et al., 1994). All mice (except *Pdgfd*) were backcrossed to C57BL/6J for at least ten generations. Genotyping was according to earlier publications. Primers for *Pdgfd* PCR were 5′-GAATCCACGTCAACCTGTTG-3′, 5′-CGCACAGGAGAATGGAGACT-3′ and 5′-GTCTGTCCTAGCTTCCTCACTG-3′.

### Tissue processing

Embryos and newborn mice were sacrificed by decapitation. Dissected organs were washed in PBS and fixed in 4% paraformaldehyde (PFA) at +4°C overnight. Mice older than one week were perfused through the heart with Hanks' balanced salt solution (HBSS) and 4% PFA before dissection, and postfixation overnight. Fixed organs were either; dehydrated and embedded in paraffin, vibratome sectioned, or soaked in 30% sucrose and embedded in OCT for cryosectioning. Spines for skeletal preparation were fixed in 95% EtOH. Meninges (for histological analyses) were peeled off from the dorsal, cerebral hemispheres of fixed and stained brains.

### Skeletal preparations, X-gal and immunofluorescence staining

Skeletal preparations of cartilage and bone were stained with Alizarin Red and Alcian Blue, (according to protocol in Behringer et al., 2014). X-gal staining on freely dissected whole brains has been described before (Andrae et al., 2014). Paraffin-embedded tissue was used to obtain a good morphology of X-gal stained tissues. Sections of 7 µm were counterstained with Nuclear Fast Red (N3020, Sigma Aldrich). For immunofluorescence staining, vibratome or cryosections were used, to better preserve the GFP expression. Sections were blocked in 1% BSA/0.5% Triton X-100 in PBS at +4°C overnight, incubated with a primary antibody (1:100) in 0.5% BSA/0.25% Triton X-100 in PBS at +4°C overnight, washed in PBS, incubated with a secondary antibody at +4°C overnight, washed in PBS and mounted in ProLongGold with DAPI (Invitrogen). Antibodies: rabbit-anti-mouse-collagen IV (AbD Serotec, 2150-1470); rat-anti-mouse-CD31 (Pharmingen, 553370); rabbit-anti-fibronectin (Sigma, F3648); rabbit-anti-laminin α1 (#317, kind gift from Prof. Lydia Sorokin, Münster, Germany); mouse-anti-NeuN (Chemicon, Mab377).

### Gene expression profiling by microarray

Cerebral meninges covering the brains of newborn pups were freely dissected and stored in RNAlater^®^ (Ambion). RNA was isolated using RNeasy micro kit (Qiagen) and quality checked in a 2100 BioAnalyzer (Agilent Technologies, Santa Clara, CA, USA). The Bioinformatics and Expression analysis core facility, Karolinska Institute, Sweden (http://apt.bea.ki.se) performed transcription profiling with Gene Chip Mouse Gene 1.0ST array. Affymetrix raw data was normalized using PLIER algorithms (Affymetrix Technical Note, Guide to Probe Logarithmic Intensity Error Estimation, http://affymetrix.com/support/technical/technotesmain.affx). We compared *Pdgfc^−/−^; Pdgfra^GFP/+^* mice with a control group consisting of all other littermates (*Pdgfc^+/+^, Pdgfc^+/−^, Pdgfc^−/−^, Pdgfc^+/+^; Pdgfra^GFP/+^* and *Pdgfc^+/−^; Pdgfra^GFP/+^*). One litter was used, including four mutants and nine controls. To select significantly differentially expressed genes in mutant mice we set the cut-off for expression fold change >2-fold and *t*-test *P*-value <0.05. The entire microarray data set has been deposited in the NCBI Gene Expression Omnibus database (www.ncbi.nlm.nih.gov/geo/, accession number GSE67644).

### Electron microscopy

P1 and P3 pups were anaesthetized and perfused through the heart with HBSS and EM-fix (2% paraformaldehyde, 2.5% glutaraldehyde, 0.02% sodium azide in PBS). The heads were cut off, the lower jaw and all skin removed, and the brains (still inside the skull) were postfixed in EM-fix overnight at +4°C. Coronal vibratome sections (100 µm) were cut through cerebrum and skull bone, to obtain intact meninges. Photographs of mutant brain slices were analyzed to identify areas of irregular meninges, which were used for the TEM analyses. Corresponding areas in non-mutant brains served as controls. Slices of brains with coverings were post-fixed for 2 h with 1% osmium tetroxide +1% potassium hexacyano-ferrate in 0.1 M sodium cacodylate buffer, pH 7.2. After *en bloc* contrasting with 0.5% uranyl acetate in water for 1 h specimens were dehydrated in ethanol to acetone and were infiltrated with epoxy resin (Agar 100, London Resins Co.). They were flat embedded between Aclar films and cured by heat. The previously selected surface regions were cut in a Leica Ultracut UC6 ultramicrotome (Leica Microsystems, Vienna, Austria) fitted with diamond knives at a section thickness setting of 50-60 nm. Sections collected on copper grids were counterstained with uranyl acetate and lead citrate before examination in a LEO 912AB transmission electron microscope. Digital image files were obtained with a Veleta 2×2 k CCD camera (Olympus-Soft Imaging Solutions, Münster, Germany) under the iTEM software (Olympus-SiS).

### Quantification of lung alveolar density

Paraffin sections of lungs from 5+5 *Pdgfc^−/−^; Pdgfra^GFP/+^* and *Pdgfra^GFP/+^* littermate controls (P15-P19), and from 7+7 *Pdgfc^−/−^* and *Pdgfc^+/+^* littermate controls (P19-P24) were counter stained with hematoxylin/eosin. Bright field images from three different areas of each lung were taken in a Nikon Eclipse E800 microscope at 20× magnification. For each lung, the areas with most sparse alveolar network were chosen. The number of open areas and their perimeter was quantified using the NIS-Elements BR2.3 program. Statistics were calculated with a paired Student's *t*-test.

### Quantification of measurements in brain

Measurements were taken from whole mount photos of dissected, fixed brains from 9 *Pdgfc^−/−^; Pdgfra^GFP/+^* and 14 littermate controls. The interhemispheric fissure (IHF) was defined as the midline distance where the two cerebral hemispheres were in contact with each other. To generate the angle (α) we drew a line from the frontal end of the IHF to the most lateral point of the brain. Statistics were calculated with an un-paired *t*-test.
